# Historical insect disturbance maps from 1985 onwards for Canadian forests derived using earth observation data

**DOI:** 10.1038/s41597-025-06269-x

**Published:** 2025-11-25

**Authors:** Pauline Perbet, Luc Guindon, David L. P. Correia, Omid Reisi Gahrouei, Jean-François Côté, Martin Béland

**Affiliations:** 1 Natural Resources Canada, Canadian Forest Service, Laurentian Forestry Centre, Quebec, QC G1V 4C7 Canada; 2 Digital Forest Lab, Faculty of Forestry, Geography and Geomatics, Laval University, Quebec City, Quebec G1V 0A6 Canada

**Keywords:** Forestry, Computer science, Imaging and sensing

## Abstract

Despite the major impact of insect outbreaks on the Canadian boreal forest and its significance in carbon monitoring, current monitoring efforts primarily rely on costly and subjective aerial survey interpretations. While satellite remote sensing has been widely used to map wildfire and harvesting disturbances, no consistent, long-term dataset exists for severe canopy loss events in coniferous forests. This paper presents the development and evaluation of annual maps of boreal forest insect pest severe disturbances in Canada from 1985 to 2024. We introduce a methodology that leverages Landsat imagery with a 30 m spatial resolution to provide a standardized, long-term record of severe pest-related defoliation. The overall prediction accuracy between the aggregated moderate and severe pest and non-pest classes was evaluated as 90%. This historical dataset offers valuable insights for forest ecology and disturbance monitoring and research, forest carbon modeling, and forest management.

## Background & Summary

Insect pests are one of the major boreal forest natural disturbances across Canada. In 2021 alone, they affected 10.7 million hectares of forest across the country accounting for 74% of all forest disturbances^1^. There are two principal types of pests in the Canadian forest that cause high rates of tree mortality: defoliators, namely the spruce budworms (*Choristoneura fumiferana* and *C. occidentalis)*, jack pine budworm (*C. pinus pinus*), and Hemlock looper (*Lambdina fiscellaria fiscellaria)*, as well as bark beetles, like the mountain pine beetle (*Dendroctonus ponderosa*) and spruce beetles (*D. rufipennis*). Defoliators feed directly on tree foliage (needles, leaves), and their impacts range from growth reduction to complete tree mortality, with responses varying from gradual decline to abrupt change^2^. By contrast, bark beetles are wood-boring insects typically associated with blue stain fungi^3^. Their infestations cause tree death, with needle discoloration progressing from green to yellow to red, followed by canopy loss^4^. Insect activity follows a cyclical pattern that can shift Canada’s forest carbon budget from a sink to a source during peak outbreaks^5–7^. Moreover, climate change may influence the frequency and spatial distribution of insect outbreaks across Canada^8,9^. This emphasizes the need to understand and to take into account the spatial extent and temporal dynamics of pest outbreaks for carbon stock calculations^10^, cumulative effects analysis^11^, and forest management planning^12^. However, a consistent, repeatable, national-scale dataset for Canada is still lacking^13^.

The most common and comprehensive method for surveying yearly pest damage at the landscape scale is aerial surveys. As forest management is governed at the provincial and territorial level in Canada, aerial surveys are conducted separately in each jurisdiction and are limited to managed forests, which excludes large tracts of northern forests and certain national parks. Consequently, the surveys are spatially and temporally heterogeneous, their methodology varies between provinces/territories^2^, and are known to suffer from interpretation bias^14,15^. Canada agglomerates these various datasets and summaries pest information at the national level through the Pest Strategy Information System (PSIS)^16^ and within the National Forestry Database (NFD)^17^. The former includes historical forest pest survey data collected by Federal and Provincial pest management agencies. The latter includes annual forest pest statistics gathered from the Federal and Provincial agencies. In comparison, remote sensing can provide cost-effective and consistent mapping using repeatable methods, which can be beneficial for large-scale studies, such as forest carbon analyses^10^.

The use of remote sensing imagery to map the occurrence or estimate the severity of pest damage has been largely studied at the local scale in Canada^2^, and in pilot regions across the globe^15^. Since the consequences of pest outbreaks are often more gradual and less often stand-replacing compared to wildfires and harvesting, they have distinct spectral-temporal characteristics that help distinguish them from other disturbances^18^. These differences can be effectively leveraged using Landsat-based temporal segmentation approaches to detect insect pest outbreaks. For example, LandTrendr (Landsat-based detection of Trends in Disturbance and Recovery)^19^ has been applied to characterize insect pest disturbances in Oregon (USA)^20^, and British Columbia (Canada)^15^. Similarly, the CCDC (Continuous Change Detection and Classification) algorithm^21^ has demonstrated strong potential for capturing defoliation patterns in regions such as Alaska, western Canada^22^ and Italy^23^. Despite the availability of nationwide disturbance maps for Canada derived from remote sensing^24–26^, existing national maps do not include pest impact information. A few studies in Canada have incorporated pest outbreaks into a distinct class^15,22,27,28^, but they are spatially limited and target a restricted number of species. The spectral effects of each pest type vary depending on the host species and environmental conditions, which constrains model transferability^29^. Additionally, low defoliation intensity results in subtle spectral changes, increasing classification errors and confusion in remote sensing-based methods^30^. Another challenge arises, within a 30 m pixel, from the fact that defoliation does not affect all trees within a pixel uniformly or at the same time^31^, particularly in mixedwood forests^2^.

Recent deep learning methods have shown strong potential for handling time-series classification in the case of agricultural crop types^32,33^, land-cover mapping^34–38^ and forest disturbance detection^28,39,40^. In a previous study, Perbet *et al*.^28^ demonstrated that TempCNN and Transformer models effectively detect spruce budworm and hemlock looper outbreaks using temporal subsequences of 5, 7, or 10 years from summer Landsat composites of boreal forest across eastern Canada. It also allows for precise classification of wildfires and clear-cut, and moderate accuracy in identifying windthrow events. The subsequence approach is particularly valuable for operational monitoring, as it allows for annual updates with minimal computational effort and better efficiency to detect gradual disturbances. In addition, the use of temporal subsequences improves the ability to distinguish overlapping disturbances. The approach of Perbet *et al*.^28^ primarily detects severe and moderate cumulative defoliation, identifying areas with heavily defoliated or dead trees, while potentially missing early or light- to moderate -stage disturbances.

In this paper, we introduce 30 m resolution annual time series maps of forest disturbance focused on insect pest impacts from 1985 to 2024. To take advantage of the model’s classification performance and produce a comprehensive, up-to-date disturbance history for Canada, wildfires, harvests, and windthrow, which represent three other major disturbances, were also mapped. We focused exclusively on insect pests affecting coniferous trees, as deciduous species are generally more resilient to defoliation and are typically affected over smaller areas^41^. We first detail the training data sampling and modeling processes, followed by an assessment of map accuracy using multiple reference sources at different scales, including ground measurements at the plot level, and photo-interpreted imagery. Additionally, we analyze results based on severity, highlighting key findings and limitations.

## Methods

### Study area

**Fig. 1:**
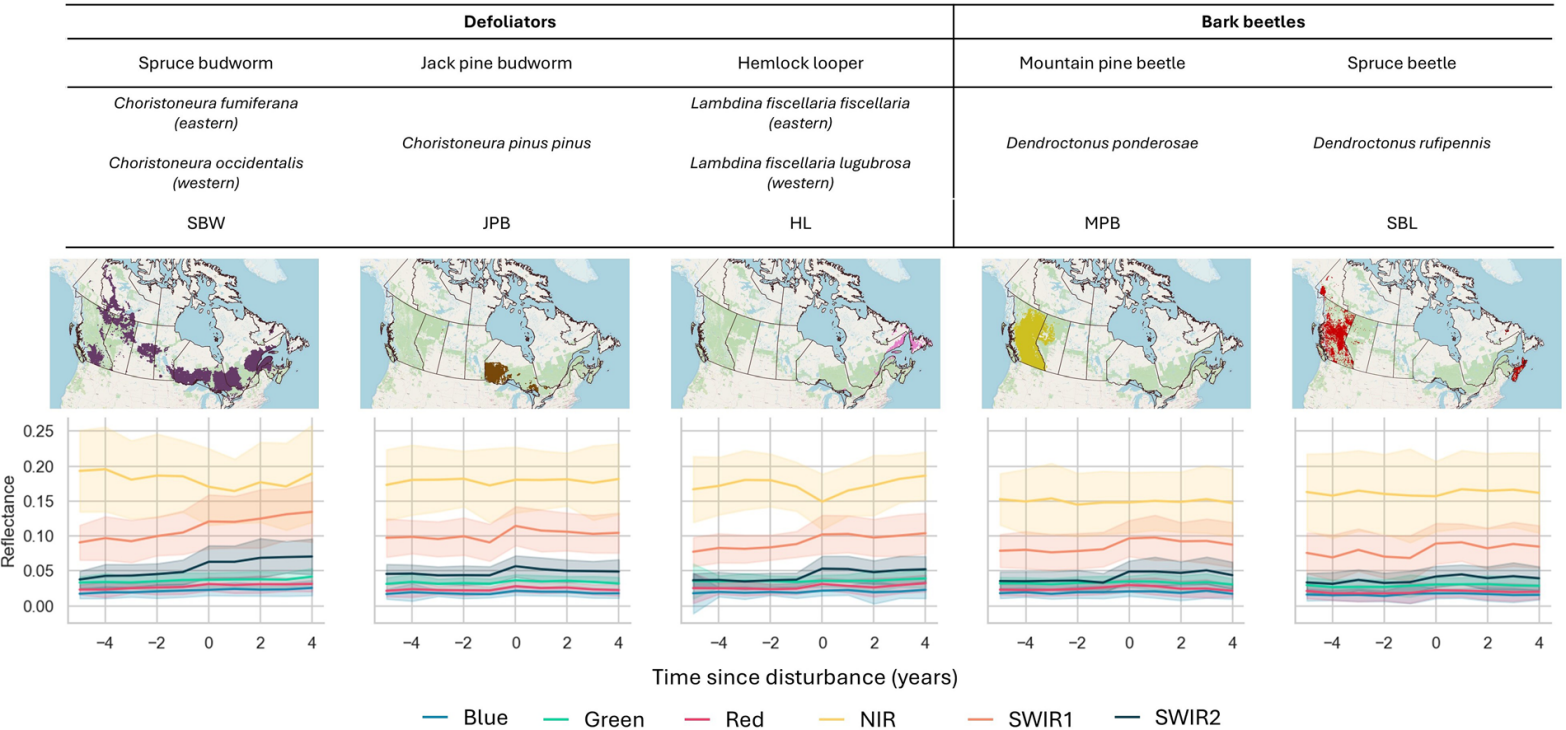
Illustration of the location of targeted pest outbreaks based on aerial surveys. The six coloured lines (Blue, Green, Red, NIR, SWIR1, and SWIR2) represent the median summer Landsat temporal trajectory of six spectral Landsat bands calculated from 500 random training points. Year 0 corresponds to the first year of insect detection in aerial surveys.

To generate a consistent national-scale record of insect pest disturbances in Canadian forests, we produced remote sensing-derived maps covering 1985–2024. These maps span 909.4 Mha of land, of which 367 Mha is classified as forest. In this study, we targeted the most damaging insect pests of coniferous trees in the Canadian forest^42^. The targeted insect species can be grouped into three categories based on their needle loss dynamics: (i) *Rapid defoliation progression* (e.g., hemlock looper), characterized by rapid defoliation within a single year; (ii) *Intermediate defoliation progression* (e.g., mountain pine beetle, jack pine budworm, and spruce beetle), where canopy loss occurs over three to five years. In the case of spruce and mountain pine beetle, the defoliation is caused by tree mortality, however, these disturbances were included in this category because the spectral change occurs gradually^20^; (iii) and *Gradual defoliation progression*, prolonged disturbances spanning five to fifteen years, as observed with spruce budworm^2,41^. Figure 1 illustrates the aerial survey estimated geographic distribution and Landsat spectral-temporal characteristics of each targeted pest species.

### Dataset

#### Training dataset

Since deep learning models require significantly larger datasets to achieve high efficiency compared to traditional machine learning methods, data preparation was a critical aspect of our study. At the pan-Canadian scale, substantial effort was dedicated to compiling and filtering input data to ensure high-quality training data that improves model performance. To ensure the model can detect subtle changes, such as defoliation, it was trained on a wide range of both disturbed and undisturbed classes. The reference dataset used for training and testing was compiled from multiple sources. The following section describes the classes, their data sources, and the criteria used to select reference points (Table 1).

**Table 1:** Classes used to train the TempCNN model.

	Before change	After change	Classe name	Source	n training	Classes in final maps
Gradual Change	Forest	Pest	Low defoliation	Aerial Survey	44235	Low defoliation
	Forest	Pest	Medium defoliation	Aerial Survey	35013	Medium defoliation
	Forest	Pest	High defoliation	Aerial Survey	23499	High defoliation
Abrupt change	Forest + No-Forest	Fire	Fire	Photo + Forest inventory	50000	Wildfire
	Forest	Total Harvesting	Total Harvesting	Photo + Forest inventory	50000	Harvesting
	Forest	Partial Harvesting	Partial Harvesting	Photo + Forest inventory	17165	Harvesting
	Forest	Windthrow	Windthrow	Photo + Forest inventory	20906	Windthrow
Undisturbed	Forest	Forest	Forest	NFI PP	50000	Undisturbed
	No-Forest	No-Forest	No-Forest	NFI PP	50000	Undisturbed
	Rocks	Rocks	Rocks	NALCMS	50000	Undisturbed
	Crops	Crops	Crops	NALCMS	50000	Undisturbed
	Urban	Urban	Urbain	NALCMS	50000	Undisturbed
	Water	Water	Water	NFI PP	50000	Undisturbed
Uncommon classes	Forest	Crops	Crops change	*ST(Forest + Crops)	50000	Undisturbed
	Forest	Urban	Urban change	*ST(Forest + Urban)	50000	Undisturbed
	Forest	Water	Dams	*ST(Forest + Water)	50000	Water extension
	Pest	Fire	Pest _fire	*ST(Pest + Wildfire)	10000	Wildfire
	Pest	Harvesting	Pest_harv	*ST(Pest + Harvesting)	10000	Defoliation followed by harvesting

The sources and references are described in the text. Photo refers to the photo-interpreted dataset from Guindon *et al*.^1^, NALCMS corresponds to the national land cover dataset^43,44^, NFI PP indicates NFI photo-plot^52^ and Forest inventory indicates data from the Quebec provincial forest database^50^. *For uncommon classes, the data is based on the synthetic combination of two classes noted as ST(classe1 + classe2).

For each extracted pixel, we generated a ten-year subsequence of six Landsat spectral bands^28^. For classes representing change, the change event was randomly positioned within the subsequence, ensuring that it did not occur in the first year. This guarantees that the model is always exposed to a healthy forest state at the beginning of the sequence^28^.

##### Aerial surveys for insect pest classes

**Fig. 2:**
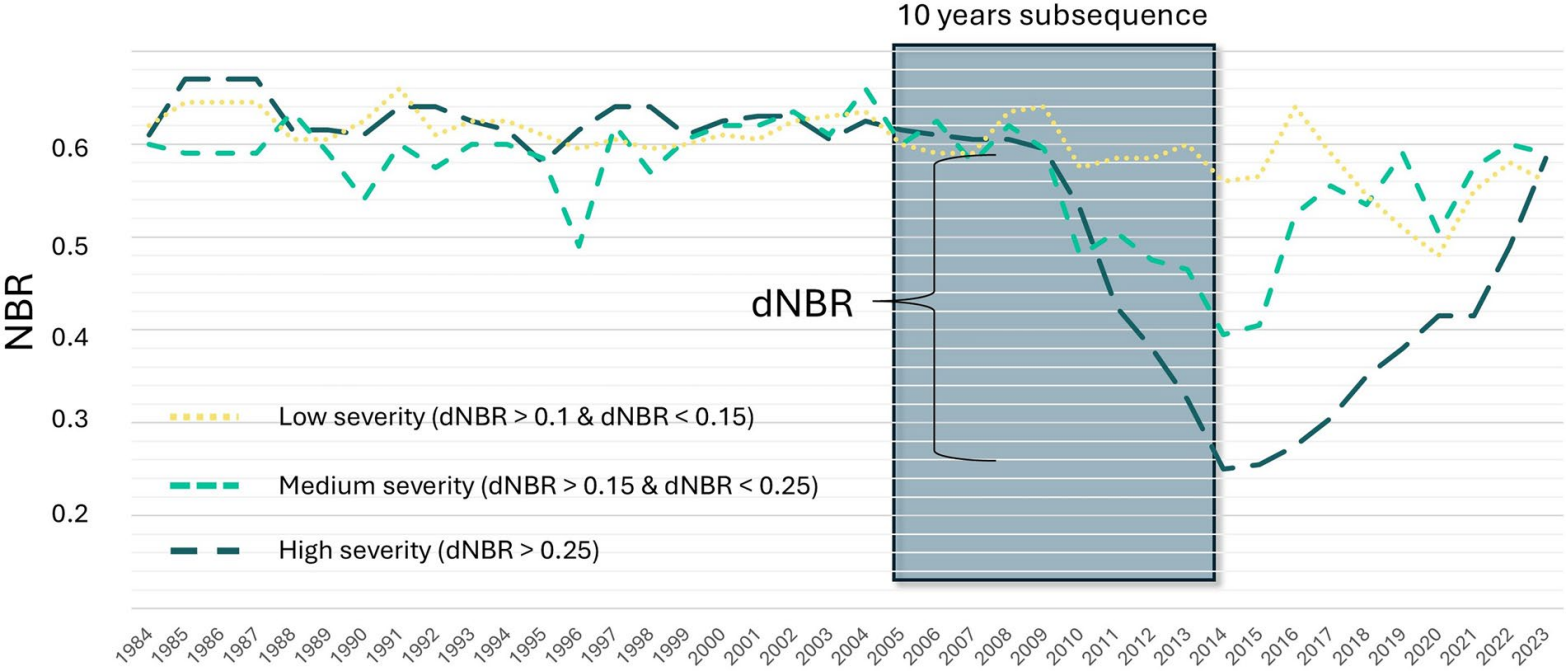
Illustration of the threshold for classifying severity based on the dNBR drop in the Landsat time series. From the ten-year NBR subsequence selected from the aerial survey, we calculated the dNBR and used it as a discriminator for the severity classes.

Training points of insect pest were randomly selected from a national polygon dataset compiled from aerial surveys between 1985 and 2022 conducted by all provinces and territories as part of pest monitoring programs. Aerial surveys are conducted to categorize current-year defoliation into severity classes (typically light: 11–30%; moderate: 31–70%; and severe: 71–100%). As aerial surveys tend to overestimate affected areas^2^, we selected pest training points within these areas according to the following strict criteria: (i) only polygons classified as medium or severe current-year defoliation were used; (ii) polygons were limited to a maximum size of 10,000 ha, as larger units were frequently found to encompass unaffected pixels; (iii) pixels overlapping urban areas, croplands, harvested areas, or burned areas were excluded using the North American Land Change Monitoring System (NALCMS)^43,44^ and CanLaD datasets^25^; and (iv) to ensure training points were located within coniferous forest, only pixels with at least 50% tree cover^45^, and classified as coniferous or mixed forest in the NALCMS^43,44^ were considered. Finally, we used a normalized burn ratio (NBR)^46^ time series to classify the spectral severity of the pest-affected pixel. NBR has proven to be sensitive to the detection of total^19,47^ and non-stand-replacing disturbances in Landsat time series^28,48^. From each selected pixel, we acquired the 10-year NBR subsequence with disturbance events occurring at the midpoint (5^th^ year). From this NBR sequence, we detected NBR decrease (dNBR), where dNBR represents the difference between two subsequent years in the sequence. If a dNBR decrease lower than 0.1 was observed, the pixel was excluded from the reference dataset. The threshold of 0.1 was determined based on our expertise and on a previous study^49^ in order to minimize potential artifacts while still detecting low severity defoliation. We then derived three classes of severity based on the dNBR decrease as: (i) low severity when dNBR was greater than 0.1 and lower than 0.15; (ii) medium severity when dNBR was greater than 0.15 and lower than 0.25; and (iii) high severity when dNBR was greater than 0.25 (Fig. 2).

### Sources for non-insect classes

#### Quebec Forest Inventory

The operational forest inventory of the Quebec provincial government (eastern province in Canada), offers a collection of databases^50^ documenting all recorded disturbances since the 1970s, which were mapped using a combination of aerial imagery, satellite imagery, and field data. From this dataset, we randomly extracted training points for wildfires, total and partial harvesting, and windthrow, applying a 30 m inner buffer.

#### Photo-interpreted data

The Canadian National Forest Inventory^51^ is a probability sample using remote sensing plots (“photo plots”) that cover approximately 1% of the Canadian land mass. This program collects high-resolution imagery in 2 × 2-km plots every 20 km over Canada’s non-arctic ecozones^52^, which are then photo-interpreted to provide reliable forest and land cover information since 2000. The resulting polygons were used to randomly extract training points for the undisturbed control classes: forest, non-forest, and water classes. To complete the disturbance training dataset, photo-interpreted samples of harvesting, wildfire and windthrow from the previous CanLaD version were incorporated^1^.

#### Other remote sensing-based products for non-forested area

To minimize confusion between pest-related changes and other land cover changes, existing land cover maps were used to randomly extract training points. The NALCMS^43,44^ was used to identify and extract training pixels for the urban, crops, and rocks classes.

#### Synthetic signature for uncommon disturbances classes

To incorporate change classes for which no extensive datasets were available, we generated synthetic spectral trajectory subsequences. For crop, urban and road conversion, and water body expansion (mostly due to damming), we concatenated a 10-year sequence of healthy forest followed by a 10-year sequence of the target land cover class. Additionally, we aimed to enhance the detection of specific cases where pest outbreaks overlapped with other disturbances, such as wildfires and salvage logging. To address this, we created synthetic classes for pest-followed-by-fire and pest-followed-by-harvesting, combining pest outbreak subsequences with corresponding fire or harvesting spectral trajectories. Subsequently, the random ten-year subsequences required for training were extracted from these synthetic spectral trajectories. Based on our initial observations, we decided not to include crop, urban, and road conversions, treating them instead as undisturbed areas. Additionally, due to high confusion, pest-followed-by-fire events were reclassified as wildfires.

#### Landsat time series

A summer Landsat composite annual time series of Canada was prepared using the pixel with the best opacity^53^ from the Landsat Collection 2 Tier 1 image collection^54^, downloaded from Google Earth Engine^55^. Multiple Landsat sensors were used to produce these composites: (i) from 1984 to 2013 Landsat 5 with Multi Spectral Scanner (MSS) was used; (ii) from 1999 onwards Landsat 7 with Enhanced Thematic Mapper Plus (ETM+); (iii) from 2013 onwards Landsat 8 with the Operational Land Imager (OLI); and (iv) from 2021 onwards Landsat 9 with the OLI sensor.

Clouds and shadows were initially removed using the quality assessment band^56^ and the best pixel was selected based on either the opacity layer for Landsat 5 and 7, or the aerosol-based ranking in the QA band for Landsat 8 and 9. Residual clouds and cloud shadows not detected by the USGS QA bands were then removed using an additional cloud/shadow mask generated by a xgboost^57^ model in R. The summer composite is based on surface reflectance bands for July and August (blue, green, red, NIR (near-infrared), SWIR1 (shortwave infrared), and SWIR2).

### Hybrid approach: the LandTrendr and TempCNN model design

**Fig. 3:**
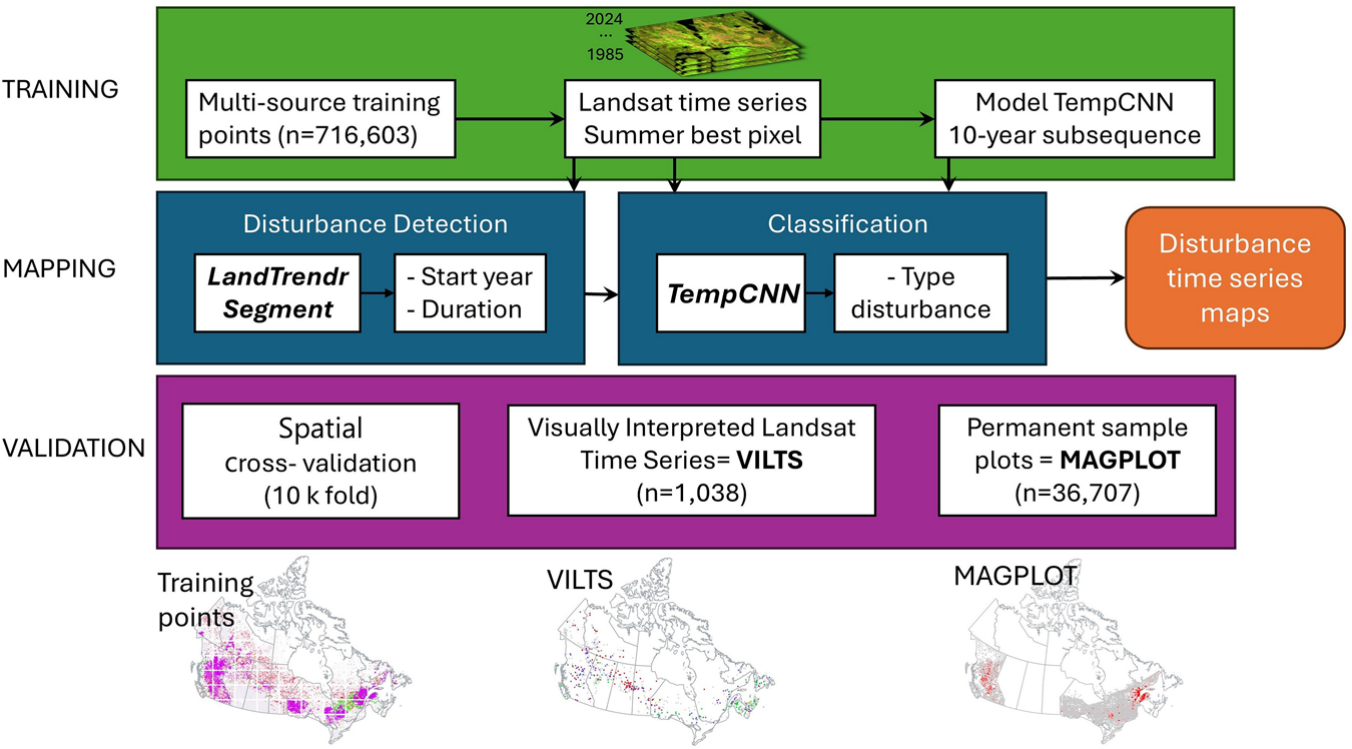
Flowchart of the methodology used in this study. MAGPlot refers to Multi-Agency Ground Plot, and TempCNN stands for Temporal Convolutional Neural Network.

The fixed ten-year window classification approach with TempCNN used in our previous work^28^ is somewhat sensitive to spectral artifact in the time series. This issue is particularly important at the end of the time series, where undetected clouds and shadows may lead to false detections. To address this limitation, we improved the original method of Perbet *et al*.^28^ by adjusting the starting year of the subsequence based on disturbance breaks identified by LandTrendr. The model design is thus divided in two steps, starting with LandTrendr disturbance detection, followed by TempCNN disturbance classification (Fig. 3).

Step 1 – Disturbance Detection: LandTrendr is a segmentation method developed by Kennedy *et al*.^19^ to detect forest disturbance breaks in a Landsat time series using spectral indices^15,20,58^ or to produce synthetic time series^53,59^. In the first step of our approach, we used LandTrendr to detect the start and end of the potential disturbance segment. The starting year of the disturbance corresponds to the first year when the NBR decrease exceeds 0.1. The ending year of the disturbance is the last year of the corresponding segment. From the entire Landsat time series (1984–2024), we extracted the 5 most recent LandTrendr disturbance segments for each pixel. We only considered up to five decrease segments up to 2024, as a sixth break occurs in only 0.002% of Canada, and more than three overlapping disturbances are extremely rare within this 40-year period^25^. For each pixel and for each disturbance sequence lasting less than 10 years, we applied the classification step to attribute the type of disturbance.

Step 2 – Disturbance type classification: Temporal Convolutional Neural Network (TempCNN) is a one-dimensional convolutional network model adapted for pixel-based temporal analysis of remote sensing data. It has been successfully applied in agricultural crop^32,33^ and forest disturbance classification^28^. In this study, the input data are 10-year subsequences of 6 Landsat spectral bands: blue, green, red, NIR, SWIR1, and SWIR2. A ten-year subsequence was considered sufficiently long to capture multi-year defoliation, yet short enough to limit overlapping disturbances. The following model parameters, selected based on previous work by Perbet *et al*.^28^, include a kernel size of 3, hidden layer dimension of 64, and dropout of 0.4. Moreover, the model was trained for 50 epochs with a batch size of 64, weight decay of 1e^−5^ and a decreasing learning rate of 0.001 every epoch. To fine-tune the model and extract performance metrics, we separated the training points using a 66 tile regular grid across Canada, to apply spatial cross-validation^60–62^. The model was trained 10 times, with each iteration using 90% of the tiles for training and 10% for testing. To evaluated potential overfitting, four tiles were reserved for validation. The final model was trained using all available points. The TempCNN model was developed and trained on a workstation equipped with an NVIDIA RTX 2080 Ti GPU.

**Fig. 4:**
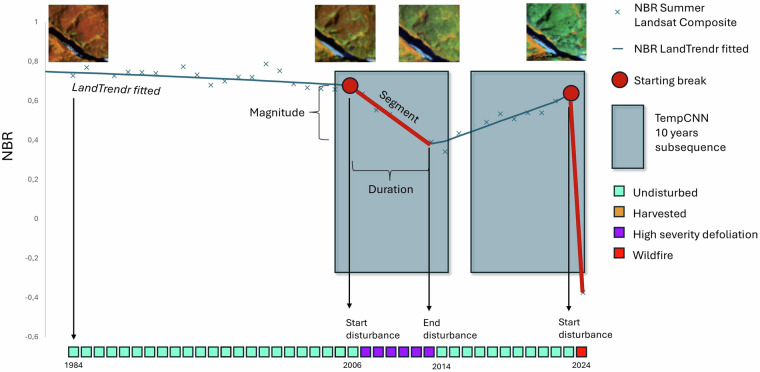
Illustration of the pixel-based method for detecting and classifying disturbances. From the Landsat NBR time series (blue x symbol), LandTrendr detects two starting break points (red points) of disturbance segments (red lines). A 10-year Landsat subsequence is extracted for each starting break point, which is then passed unto the TempCNN model for disturbance classification. Using the start and the end years of the LandTrendr segment and the classes predicted by TempCNN, a disturbance time series is created, with the type of disturbance for each year. In the case of rapid disturbances (wildfire, harvesting, windthrow), only the first year of the disturbance is retained in the time series. For pest disturbances, the information is preserved throughout the duration of the event, as defined by the LandTrendr segmentation.

The TempCNN model was applied on a pixel-based 10-year subsequence, starting from the year before the LandTrendr-detected disturbance begins (Fig. 4). This allows TempCNN to capture the entire sequence of the disturbance effect on the spectral values, as well as any potential recovery effects. If the first year of the subsequence corresponds to missing data (due to clouds, shadows, or artifacts), the first available year with spectral information was used instead. For all breaks starting after 2015, the subsequence begins in 2015, as the model is trained on 10-year subsequences and can only operate with a full 10-year range.

Using the TempCNN predicted disturbance type and the LandTrendr segment start and end years, we were able to create a yearly, pan-Canadian disturbance time series at the pixel level. When the predicted disturbance type is known to be rapid and stand-replacing, such as wildfire, total harvesting, windthrow, or water expansion, the disturbance is indicated for only one year in the time series. For wildfire classes, if missing data cause the LandTrendr segment to exceed more than 2 years, the starting year is set to the end year of the segment. In the case of multi-year defoliation, such as that caused by insect pests, the predicted type of disturbance is preserved throughout the duration of the disturbance segment, as defined by LandTrendr. The resulting time series was then used to perform the analyses described in the rest of this study.

The creation of the historical disturbance maps was made possible using the Government of Canada’s High-Performance Computing (HPC) service. The country was divided into approximately 2,000 tiles of 10,000 km² each to enable parallel processing. The complete workflow, from LandTrendr break detection to map production, takes approximately three days.

## Data Records

The resulting maps are available on the Government of Canada open-data platform (10.23687/902801fd-4d9d-4df4-9e95-319e429545cc)^63^. This project is in line with the original machine learning-based CanLaD initially developed nearly 10 years ago which did not include insect pest disturbance. The dataset presented here that was developed with a more powerful algorithm will replace the original CanLaD^1,25^ harvest and fire time series maps, and could be complemented with older disturbance data^64^.

The dataset includes:

In the parent folder: (i) the cartographic projection definition in WKT format (_projectionDefinitionWKT_lcccan83.prj), (ii) a raster containing the last Julian day of the time series, expressed as the number of days since January 1, 1970 (canlad_JJ_max.tif), (iii) two readme files in English and in French (_lisezmoi.txt and _readme.txt), (iv) a csv file with legend name and color of the disturbance types (legend_type.csv).

In the Disturbances_Latest folder: a combined raster of the latest disturbance type (canlad_1985_2024_latest_type.tif), along with the associated starting year of disturbance (canlad_1985_2024_latest_year.tif) and ending year of disturbance (canlad_1985_2024_ending_year.tif). For each raster, the corresponding pyramid file (.ovr), auxiliary information file (.aux.xml), and QGIS style file (.qml) are also provided.

In the Disturbances_Time_Series folder: annual rasters of disturbance type from 1985 to 2024 (canlad_annual_*.tif).

Raster class codes are: 1 = Wildfire; 2 = Harvesting; 3 = Windthrows; 4 = Water extension; 5 = Defoliation-followed-by-Harvesting; 6 = Low severity defoliation; 7 = Medium severity defoliation; 8 = High severity defoliation. The QGIS style file is available in the repository, as well as a csv file linking disturbance class codes and labels.

To reduce noise and correct classification artifacts, several post-processing steps were applied to the final maps. First, isolated disturbance pixels were removed using a 12-pixel sieve filter, with the sieve mask created based on a five-year moving window (affecting approximately 10% of the disturbed pixels). Second, pixels classified as harvesting or pest outbreaks inside wetlands determined according to the forest land cover class from the Spatialized Canadian National Forest Inventory (SCANFI)^53^, were removed. This filter affected approximately 2% of the disturbed pixels.

The data is licensed under the Creative Commons Attribution 4.0 International license.

## Technical Validation

Two independent spatial-temporal validation analyses were carried out to assess the performance of the data product described in this study. First, we used a series of available permanent sample plots for validation across three provinces. Since these plots have restricted temporal (e.g., a specific year of measurement) and spatial range adapted for disturbance detection and classification, they could not be used to validate all our disturbance classes across all provinces and disturbance duration. To complement this, we developed an independent validation dataset by visually photo-interpreting pixel disturbance classes. Both validation efforts were conducted using the raw annual disturbance time series maps, prior to any post-processing. To reduce the influence of isolated pixels on performance metrics, all statistics were based on the majority class, comprising the 9 surrounding pixels around the validation points.

### Multi-agency ground plot validation

We used the Multi-Agency Ground Plot (MAGPlot)^65^ database version 1.1 which is the Canadian forest ground-plot data repository that harmonizes contributed provincial and territorial permanent forest sample plots across Canada. Plots within this database typically cover an area of 400m^2^ and include individual tree species measurements and health conditions. We selected plots from the provinces of Ontario, British Columbia, and Quebec based on strict quality criteria, namely accurate plot geolocation, availability of defoliation data, and overall data abundance.

For each of the 52,712 ground plot measurements since 1985, we estimated the proportion of aboveground biomass (AGB) associated with trees impacted by defoliators in a plot, referred to as %AGB defoliated. To do this, the AGB of trees with a diameter at breast height (DBH) greater than 9 cm was individually estimated using the allometric models by Lambert *et al*.^66^ and Ung *et al*.^67^, based on DBH measurement. Then, the AGB of all coniferous trees inventoried as defoliated were summed to compute the defoliated AGB. The plot-level proportion of AGB defoliated was then calculated according to Eq. (1).

1 $$ \% {AGB\; defoliated}=\frac{\sum {AGB\; defoliated}}{\sum {AGB\; total}}\times 100$$

**Fig. 5:**
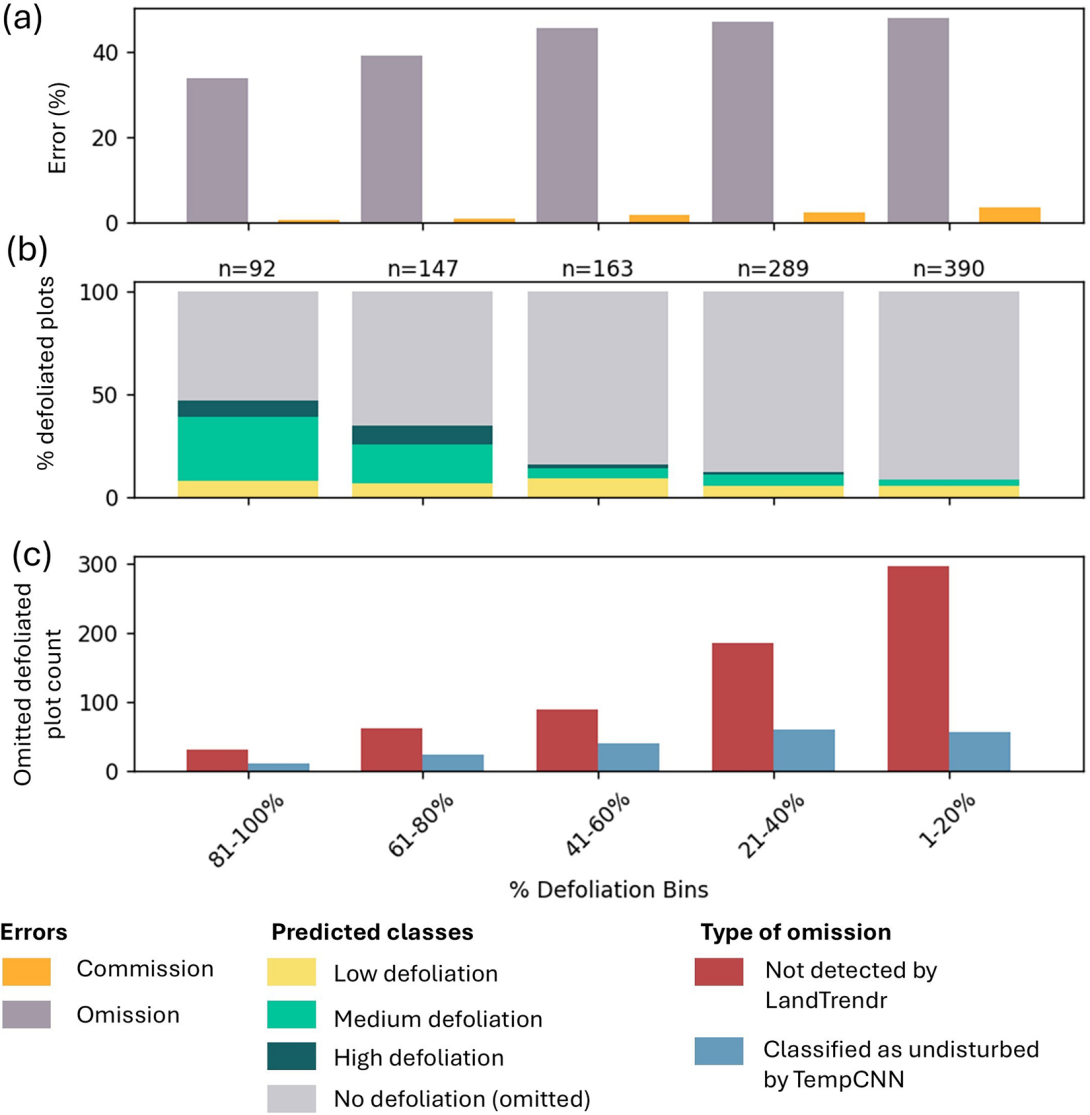
Validation using ground plot (MAGPLOT): (**a**) Omission and commission errors in defoliation classification, (**b**) predicted defoliation classes and (**c**) sources of omission errors per biomass defoliation percentage bins.

After applying a filtering approach (e.g., excluding deciduous defoliation, measurements with more than 80% dead trees from unknown disturbances, or plots with AGB lower than 50 t/ha), we analyzed 36,707 measurements (Quebec = 25,659; Ontario = 9,137; British Columbia = 1,914). Figure 5a illustrates the commission and omission errors as a function of the biomass defoliation percentage extracted from field data. Ground plots were grouped into 5 bins based on the percentage of AGB defoliated. Figure 5b shows the classification results of the defoliated pixels by severity category. We observed a decrease in omission errors and an increase in the number of pixels classified as medium or high severity in plots with more than 60% defoliation. These results indicate that low defoliation levels visible in the field are not well detected by the annual time series approach. The regular proportion of pixels classified as low severity across all bins suggests a weak correlation between the field-based defoliation and severity classes based on NBR drop.

A visual analysis of the 115 false detections (where defoliation was not recorded in field measurements but was detected by our model) revealed that in 70% of instances, spectral changes was visible in the Landsat time series. Additionally, 33% of these cases were also identified by aerial surveys conducted in the same year as the field visits. These results highlight the differences between field-based and remote sensing observations, since defoliation occurring in the upper canopy may not always be visible from ground-level measurements. These discrepancies also highlight the limitations of using permanent sample plots for validating remote sensing-based analyses, as they were not originally designed for defoliation monitoring.

Figure 5c shows the count of omitted detections categorized by error type. In most cases, the omission occurred because the LandTrendr approach failed to detect the target disturbance. As a result, these pixels were not processed by the TempCNN disturbance classification model. Omission is primarily related to the 0.1 NBR threshold selected for disturbance detection, which is insufficient for identifying low levels of defoliation.

### Visually Interpreted Landsat Time Series validation Points (VILTS)

In order to have an independent validation dataset that was strictly established for remotely sensed disturbance validation, we created an additional photo-interpreted dataset based on the Landsat time series, the Visually Interpreted Landsat Time Series dataset (VILTS). Based on best practices outlined by Olofsson *et al*.^68^, we used a stratified random sampling with 1,100 sampling pixels to achieve a confidence level of 95% and a standard error of 0.02 for the pest class. VILTS points were automatically selected randomly using the resulting latest disturbance maps and the aerial survey polygons to enable species-level analyses. We made sure that those points were at least 200 m away from existing training points. The expert interpreter used Landsat summer imagery, NBR time series (Temporal/Spectral profile tool in QGIS), and high-resolution imagery freely available via Google Earth Pro to determine the class of disturbance, as well as the starting and ending years of the disturbance as proposed by Cohen and al.^47^. Since the severity and type of pest are extremely challenging to rigorously assess visually, only a single pest class was assigned by the interpreter. Samples that were difficult to interpret were not included in the final 1,073 VILTS points. We considered a prediction correct if the year predicted by our LandTrendr/TempCNN model overlapped with at least one visually photo-interpreted disturbance year.

Since the sum of the undisturbed pixels in the 40-year time series is significantly larger than any individual stratum, using adjusted accuracy metrics will result in excessive omission errors^69^. While the adjusted overall accuracy will appear greater, the omission error will not be interpretable. To address this issue, accuracy metrics and the confusion matrix for all classes was reported as count values (Table 2). The overall accuracy was 81% based on photo-interpreted points. Wildfire was the most accurate class, with an omission (missing detections) error of 17% and commission (false detections) error of 2%. Harvesting showed moderate accuracy, with 31% for omission and 14% for commission error. Windthrow had a greater level of error with 55% omission and 58% commission error, indicating a considerable level of false detections. For the pest classes, we found a reasonable commission of 19% but a greater omission of 52%.

**Table 2:** Confusion matrix of the Visually Interpreted Landsat Time Series dataset (VILTS) dataset.

Maps	Reference data						
	No change	Wildfire	Harvesting	Windthrow	Pest_Harv	Pest	Commission
No change	**531**	8	31	9	2	95	21%
Wildfire	0	**116**	0	0	0	2	2%
Harvesting	3	0	**96**	3	6	3	14%
Windthrow	1	3	4	**10**	0	6	58%
Pest_Harv	0	2	3	0	**18**	0	22%
Pest	7	10	5	0	1	**96**	19%
Omission	2%	17%	31%	55%	33%	52%	
Total count	542	139	139	22	27	202	**OA = 81%**

(Pest_Harv refers to pest-followed-by-harvesting).

**Fig. 6:**
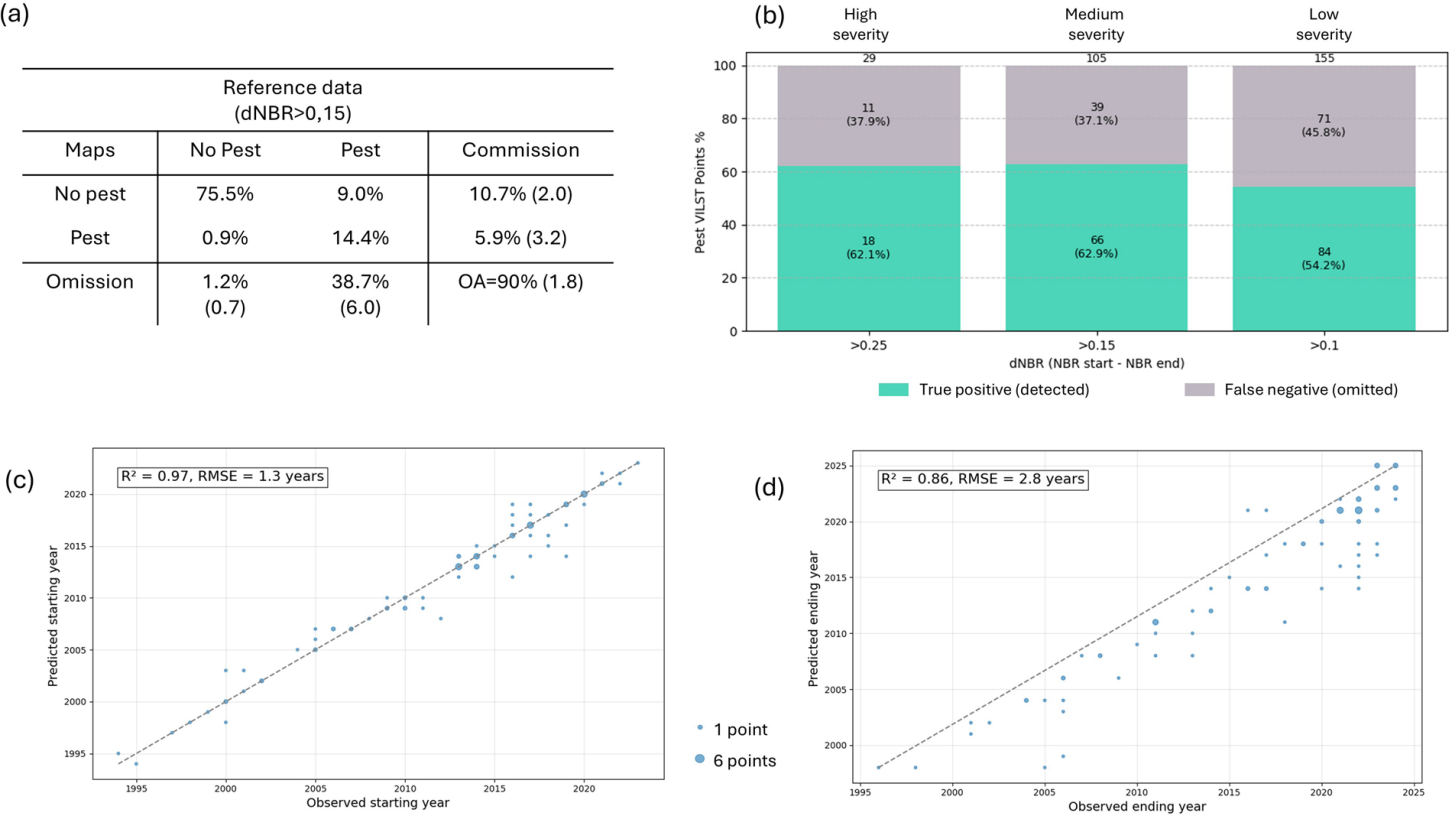
(**a**) Confusion matrix including medium and severe defoliation, with area adjusted values; standard errors are shown in brackets. (**b**) Bar plot showing the percentage of false negative (omissions) or true positive (correct prediction) for Visually Interpreted Landsat Time Series dataset (VILTS) points visually identified as pest defoliation, as a function of the dNBR. The dNBR was calculated from VILTS points between the visually estimated date of start and end of defoliation. (**c**) Scatterplot of predicted versus observed starting years for the correctly classified defoliated VILTS points. (**d**) Scatterplot of predicted versus observed ending year for the defoliated points. Point size indicates the number of overlapping VILTS points.

We followed the calculation methods of Stehman *et al*.^70^ for the aggregated pest/no-pest classes, and used stratified area estimates based on whether or not pest outbreak have been detected by aerial surveys since 1985. For medium and severe defoliation (dNBR > 0.15), the area adjusted overall accuracy was 90.0% ± 1.8%, with a commission error of 6.7 ± 3.5% and omission error of 41.0 ± 6.2% (Fig. 6a). We further analysed the percentage of omitted points based on the difference in NBR between the observed starting year and the final year of the outbreak (Fig. 6b). As expected, pixels with a small decrease in NBR (i.e. low severity outbreaks) are more frequently omitted.

Finally, temporal accuracy was assessed using the R² value between the LandTrendr-estimated date and the interpreter-observed date, for defoliated plots that were correctly detected. Figure 6c and d show strong agreement between the predicted and the photo-interpreted disturbance start (R^2^ of 0.96) and end year (R^2^ of 0.80). However, the ending year predicted was most of the time earlier than the photo-interpreted insect pest outbreak duration.

### Comparison with other national forest datasets

**Fig. 7:**
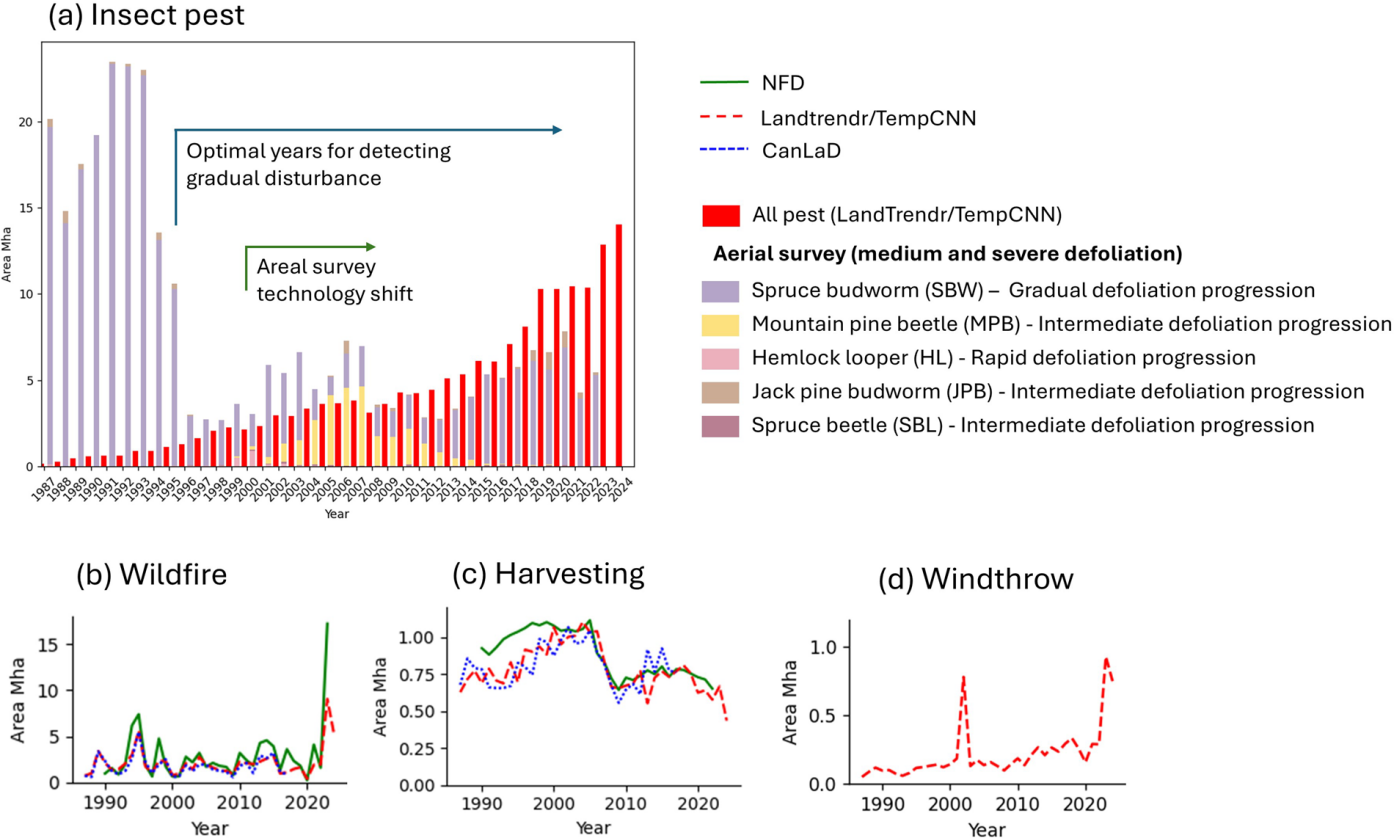
Area impacted by various disturbances over time according to our methodology (dashed red line), the National Forest Database (NFD; green line) and the Canada Landsat Disturbance data product of Guindon *et al*.^25^ (CanLaD; dashed blue line). Figures (**a**–**c**) are based on the detected disturbance starting year, while Figure (**d**) is derived from time series data, which highlights the gradual impact of pest outbreaks. As our approach requires a healthy year at the beginning of the sequence, the major spruce budworm outbreak from the 1970s to 1995 was not detected. The optimal years for detecting gradual disturbances are indicated by the blue arrow.

Figure 7 presents the impacted area over the year of the main disturbances classes compared to the National Forestry Database^17^ (NFD) between 1990 and 2022 (or 2023 for the wildfire as it was already available) for the whole of Canada. For insect pests (Fig. 7a), we present the annual aerial survey area of medium and severely defoliated tagged polygons, compared to our model predictions. Major observations highlighted by the figure include two peaks of spruce budworm activity: one from the early 1970s until 1995 (primarily in eastern Canada) and another between 2013 and 2020 (in Quebec). Additionally, there was a peak of mountain pine beetle activity between 2004 and 2008 (British Columbia). The first spruce budworm peak recorded by aerial surveys is likely overestimated due to the coarse digitization methods used prior to the adoption of onboard tablet technology^2^. Nevertheless, this peak was not captured by our model. Given the gradual spectral signature of spruce budworm outbreaks, the starting year of the outbreak predates the time series. Since our model relies on detecting changes relative to a baseline of healthy forest conditions, it failed to consistently detect this large spruce budworm outbreak. Consequently, this result suggests that reliable historical mapping of pest outbreaks using this method becomes realistic only after approximately 10 years into the time series. After 1995, the trend in area impacted by pests from our model more closely match the aerial surveys. We observed the peak of the mountain pine beetle outbreak around 2005 and the recent spruce budworm outbreak in eastern Canada.

We found a clear correspondence between our maps and the NFD for wildfire (Fig. 7b). In 2023, our model underestimated the land area impacted by fire, likely due to the temporal mismatch between this record-breaking fire season, which extended into the fall^71^, and the Landsat summer composites used. As a result, the peak in wildfire area in 2024 thus includes a part of the area affected by the 2023 wildfires. The delay of detection is a limitation of using the summer composite time series already shown in several studies^18,29^. For harvesting (Fig. 7c), the annual area disturbed is comparable only after 2005. For windthrow events (Fig. 7d), although no national database exists to validate our data, the 2003 peak aligns with a snow damage event in Ontario^72^, and the 2023 peak corresponds to the impact of Hurricane Fiona in Nova Scotia^73^. These windthrow peaks are notable as they nearly match the annual harvested area in terms of impact.

### Aerial survey visual comparison

**Fig. 8:**
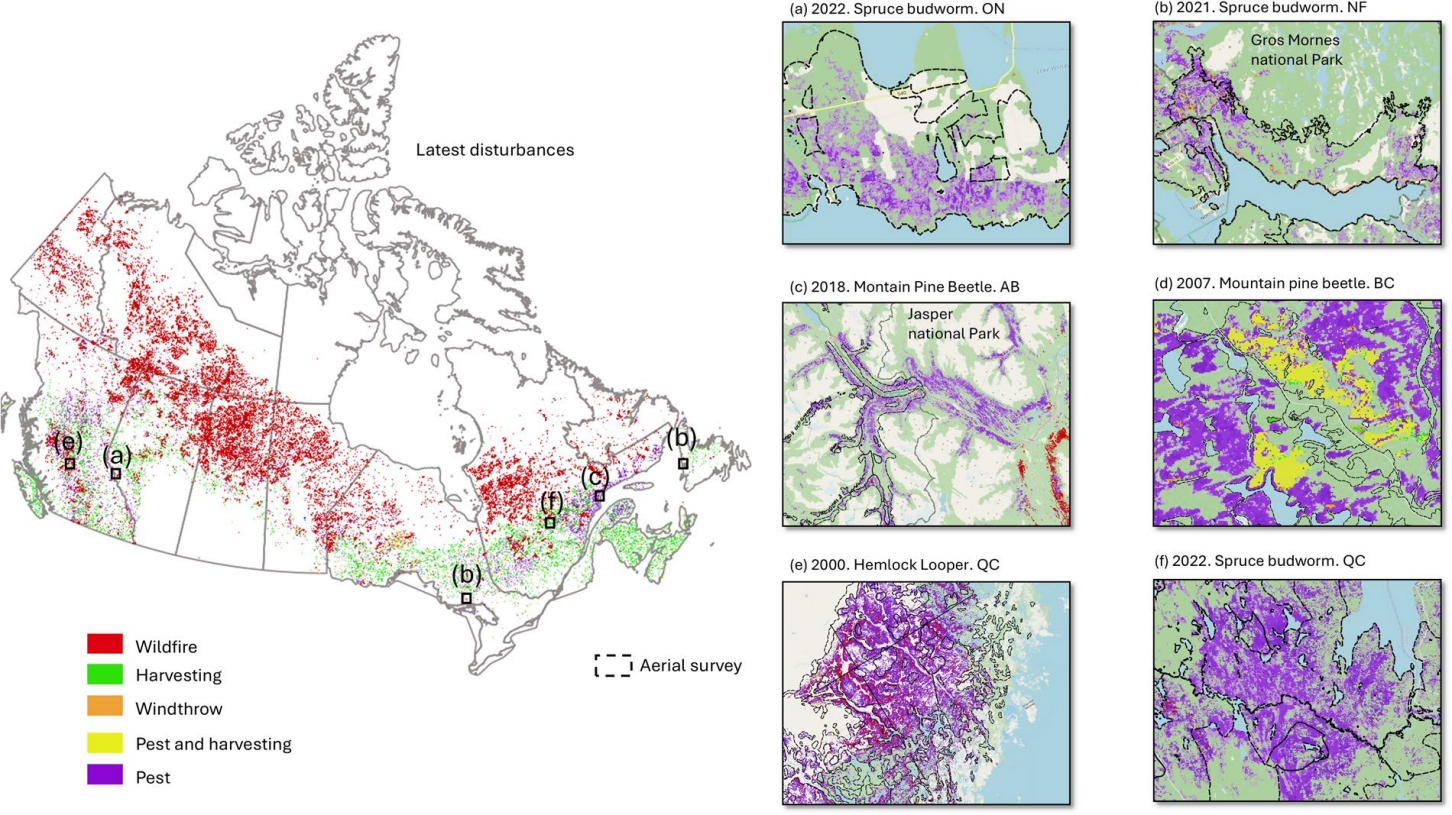
Latest disturbances map and yearly regional examples (**a** to **f**) displayed over OpenStreetMap^87^, with corresponding aerial survey polygons for the same year (in dash black outlines).

Figure 8 shows the latest disturbance maps from 1985 to 2024, along with yearly regional examples overlaid with the corresponding aerial survey polygons. Overall, our results show that aerial survey polygons overestimated the damage extent compared to the disturbance maps derived from remote sensing. For instance, in southern Ontario and eastern Newfoundland (a and b), the aerial survey includes large non-forested areas (beige color) that are correctly classified as undisturbed by our remote sensing model. In Fig. 8c, we show how this data product detects missing insect pest outbreaks outside the boundaries of existing aerial surveys (e.g. Jasper National Park). Figure 8d highlights detections of salvalge logging following severe mountain pine beetle outbreaks in British Columbia. Example 8e shows how the severe hemlock looper outbreak in Quebec, which tends to be misclassified as fire in other remotely sensed disturbance detection products, was mostly classified as an insect pest outbreak. Figure 8f demonstrates the detection of the recent spruce budworm outbreak in Quebec.

Forest disturbance is a natural part of boreal and temperate forest ecosystem dynamics, although climate is expected to have repercussions for the severity and patterns of these disturbances^74,75^. Based on this new method of detecting disturbance across Canadian forests, 36% of Canada’s forest area has been impacted by disturbance between 1985 and 2024. Of that, 19% of forest was impacted by fire, 8% due to pests, 2% due to windthrow and 8% due to harvesting activities. Disturbance has been detected at least once for 31% of the forest area in Canada, with 5% having experienced overlapping (multiple) disturbances. Disturbance has not been detected for 69% of the forest area in Canada during the analysis period.

## Usage Notes

### Uncertainty and limitations

A single national forest disturbance model ensures spatial consistency and facilitates annual map updates. However, this approach may lead to uncertainties, given the ecological variability across Canada’s regions, seasonal differences in vegetation phenology, and the distinct spectral and temporal signatures of disturbance types, especially insect pest outbreaks. Users should be aware of the following limitations when interpreting and using these maps. Even though our methodology fails to reliably detect low-severity insect pest outbreaks, national-scale carbon modeling focuses primarily on severe defoliation events that lead to reduced growth or tree mortality. As we have shown, such moderate to severe events are consistently detected by our model. Unfortunately, the 10-year analysis window, combined with the requirement that forest conditions appear healthy at the start of this period, limits our ability to detect defoliation events that occurred before 1995. Similarly, this limitation has minimal impact on potential large scale ecological applications involving pest interactions. Caution is advised when using these maps as a substitute for aerial surveys, since our model is less effective in detecting recent or low-severity defoliation events. As highlighted in previous studies using Landsat time series^59,76–78^, the end of the sequence may cause increased confusion due to the limited information available to the model regarding the disturbance recovery trajectory, hampering the model’s ability to accurately distinguish between real disturbances and spectral noise. For this reason, the most recent years in the historical maps may exhibit greater commission errors, potentially leading to misinterpretation.

### Potential applications

With an overall classification accuracy of 90% between the aggregated pest and non-pest classes, these historical Canadian disturbance maps, using a consistent mapping methodology, provide a comprehensive portrait of the importance of forest pests in Canadian forest ecosystem dynamics. These maps highlight regions where defoliation can hinder tree growth and contribute to tree mortality, providing valuable inputs for studies in forest dynamics, ecology, and carbon modeling^13^.

Furthermore, these new data can aid in understanding interactions between forest ecology dynamics and forest management activities, such as those between wildfires and pests^79,80^, moose browsing^81^, bird migration^82^, and habitat of woodland caribou^83^. In addition to traditional harvested and fire classes, the inclusion of a pest disturbance class can enhance understanding of the drivers of forest cover loss or change, particularly in analyses conducted at biome or global scales^45,84–86^.

## Data Availability

The dataset is available on the Government of Canada open-data platform (https://open.canada.ca/data/en/dataset/902801fd-4d9d-4df4-9e95-319e429545cc)^63^.

## Code availability

The code used to develop this work can be accessed through the following link: https://github.com/NRCan/CanLaD. This repository includes python script of the TempCNN model, and script used to create time series, latest maps and cleaning.
